# Sex, myelin, and clinical characteristics of Parkinson’s disease

**DOI:** 10.3389/fnins.2023.1235524

**Published:** 2023-09-13

**Authors:** Jiayue Cai, Jowon L. Kim, Yuheng Wang, Tobias R. Baumeister, Maria Zhu, Aiping Liu, Soojin Lee, Martin J. McKeown

**Affiliations:** ^1^School of Biomedical Engineering, Shenzhen University Medical School, Shenzhen University, Shenzhen, Guangdong, China; ^2^Department of Medicine, The University of British Columbia, Vancouver, BC, Canada; ^3^School of Biomedical Engineering, The University of British Columbia, Vancouver, BC, Canada; ^4^Department of Electronic Science and Technology, University of Science and Technology of China, Hefei, Anhui, China

**Keywords:** myelin water fraction, sex, white matter microstructure, clinical characteristics, Parkinson’s disease

## Abstract

**Objective:**

To determine if there are sex differences in myelin in Parkinson’s disease, and whether these explain some of the previously-described sex differences in clinical presentation.

**Methods:**

Thirty-three subjects (23 males, 10 females) with Parkinson’s disease underwent myelin water fraction (MWF) imaging, an MRI scanning technique of *in vivo* myelin content. MWF of 20 white matter regions of interest (ROIs) were assessed. Motor symptoms were assessed using the Unified Parkinson’s Disease Rating Scale (UPDRS). Principal component analysis, logistic and multiple linear regressions, and t-tests were used to determine which white matter ROIs differed between sexes, the clinical features associated with these myelin changes, and if overall MWF and MWF laterality differed between males and females.

**Results:**

Consistent with prior reports, tremor and bradykinesia were more likely seen in females, whereas rigidity and axial symptoms were more likely seen in males in our cohort. MWF of the thalamic radiation, cingulum, cingulum hippocampus, inferior fronto-occipital fasciculi, inferior longitudinal fasciculi, and uncinate were significant in predicting sex. Overall MWF and asymmetry of MWF was greater in males. MWF differences between sexes were associated with tremor symptomatology and asymmetry of motor performance.

**Conclusion:**

Sex differences in myelin are associated with tremor and asymmetry of motor presentation. While preliminary, our results suggest that further investigation of the role of biological sex in myelin pathology and clinical presentation in Parkinson’s disease is warranted.

## Introduction

Parkinson’s disease (PD) is a progressive neurodegenerative disorder characterized by dopaminergic denervation in the substantia nigra, resulting in cardinal symptoms of motor dysfunction such as tremor, rigidity, bradykinesia, and postural instability. Despite well-established observations of differences in clinical characteristics between male and female PD patients (Haaxma et al., 2007), effects of biological sex are often neglected, with an assumption of generalizability of findings to both sexes. Sex is an important factor in PD, with epidemiologic differences seen in disease prevalence, symptomatology, and natural progression between male and female patients. The greater prevalence of PD among males (male: female ratio of 2: 1) suggests the presence of inherent biological differences that increase male predisposition. Male patients generally have greater rigidity, as well as greater number of symptoms, including rapid eye movement sleep behavior disorder (Yoritaka et al., 2009), writing difficulties (Scott et al., 2000), speech problems (Scott et al., 2000), and gait problems (Scott et al., 2000). In contrast, female patients generally have a later age of onset, are more likely to present with a more benign, tremor-dominant phenotype with slower disease progression (Haaxma et al., 2007), and have less rigidity (Baba et al., 2005), but greater severity of levodopa-related dyskinesias (Colombo et al., 2015).

Sex differences in clinical presentation may be related to sex-specific differences in the brain, such as the sexual dimorphism normally seen in the overall human brain, as well as specifically in the basal ganglia. Sexual dimorphism in striatal dopamine levels, with females having significantly higher dopamine uptake, have been frequently observed in healthy adults (Wong et al., 2012), as well as in the setting of PD (Haaxma et al., 2007). Prior imaging studies of healthy individuals have found that females have larger relative gray matter volume, while males have larger relative white matter (WM) volume (Gur et al., 1999; Chen et al., 2007).

There is also emerging evidence that sex hormones play a significant role in the sexual differentiation of white matter (WM) microstructure, which may be driven by differences in myelin (van Hemmen et al., 2016). In a mouse model, male mice have thicker myelin sheaths and greater density of oligodendrocytes (Abi Ghanem et al., 2017). Female mice have greater levels of a biochemical marker of myelination in the orbitofrontal cortex (Massella et al., 2012), possibly related to differential exposure to gonadal hormones during puberty (Darling and Daniel, 2019). Several whole-brain diffusion tensor imaging (DTI) studies of healthy adult humans have found that males have higher fractional anisotropy (FA) than females in many WM regions of interest (ROIs) (van Hemmen et al., 2016; Jung et al., 2019). While FA is the most frequently reported DTI parameter, a few studies have reported that higher FA in men is actually driven by lower radial diffusivity (RD) values in the same WM ROIs (Menzler et al., 2011; van Hemmen et al., 2016), further suggesting a role for differences in myelin contributing to the sex-specific differences in WM microstructure. However, the literature on sexual dimorphism in WM microstructure in the setting of PD is currently lacking.

Microstructural changes in normal-appearing WM are being increasingly recognized in PD. Oligodendrocytes, responsible for myelination, are classically implicated in multiple system atrophy, but autoantibodies against proteins of oligodendrocytes have also been described in PD (Papuć and Rejdak, 2017). A genome-wide association study found a surprising relation between PD markers and oligodendrocytes with alterations seen at disease onset (Bryois et al., 2020), suggesting potential contribution of disordered myelination in the cellular pathology of PD. Though the causal mechanism of nigrostriatal dopaminergic denervation is not yet known, cellular pathologies such as neuroinflammation, oxidative stress, and microglial activation likely play an important role in PD pathogenesis (Ferreira and Romero-Ramos, 2018), with increased levels of pro-inflammatory cytokines and microglial activation observed in the areas most affected by dopaminergic cell death (McGeer et al., 1988). These findings, along with aforementioned sexual dimorphism in the normal human brain and basal ganglia, suggests a role for investigation of sex-specific WM pathology in PD.

Non-invasive *in vivo* assessment of brain WM is most commonly done via DTI, which is sensitive to a spectrum of white matter tissue changes, including axonal size, myelination and cellular density, but may also be influenced by other factors, such as inflammation. FA is the most commonly reported DTI measure, with low FA values often inferred to be indicative of decreased myelination and axonal degeneration. Although FA is affected by myelin, it is also sensitive to many factors within the imaging voxel, complicating its biological interpretation. Furthermore, there have been DTI studies that identified predominance of RD changes in PD (Theilmann et al., 2013), a DTI parameter that is classically thought to represent degree of myelination, suggesting that myelin may play a larger role in PD than previously attributed. While these metrics provide relatively broad quantifications of white matter microstructure, they are not specific characterizations of myelin content.

In light of increasing interest in myelin pathology in PD, more specific imaging biomarkers targeting myelin are needed. Myelin water fraction (MWF) has much higher coefficients of determination with histological samples compared to DTI (Laule et al., 2006), as well as good scan-rescan and inter-site reproducibility (Lee et al., 2018). MWF is based on T2 relaxation data, collected via a 3D gradient echo spin echo (GRASE) sequence. The T2 distribution is fitted with multiple T2 components, including a short T2 relaxation component which can be attributed to water trapped between myelin lipid bilayers. MWF is then defined as the ratio of the short T2 relaxation component to the total T2 distribution. Unlike DTI, MWF is relatively insensitive to inflammation and other non-myelin related factors.

In this study, we utilize MWF as a surrogate marker of *in vivo* myelin content. MWF profiles have recently been demonstrated to correspond to clinical features in PD such as rigidity, apathy, depression, and cognition (Baumeister et al., 2019; Cai et al., 2022). Here, we aim to extend these prior results to demonstrate biological sex differences in myelin in PD. Specifically, the goal of this study was to (1) determine if MWF in PD shows differences between sexes; and (2) examine if these differences are associated with differences in clinical presentation.

## Materials and methods

### Study subjects

A total of 33 patients (23 males, 10 females) with idiopathic PD participated in this study. All subjects provided informed written consent according to the Declaration of Helsinki prior to undergoing the MRI scan. This study was approved by the Institutional Ethics Review Board.

### Clinical assessment

All PD subjects were assessed in the medication ON state, using part III of the Movement Disorders Society Unified Parkinson’s Disease Rating Scale (MDS UPDRS-III) for symptom severity and the Hoehn and Yahr (H&Y) scale for disease staging. Motor assessments were administered by experienced research personnel who were certified by the International Parkinson and Movement Disorder Society. A summary of the clinical and demographical characteristics is available in Table 1. Clinical characteristics of male and female PD patients were compared by calculating axial, bradykinesia, rigidity, and tremor subscores of UPDRS-III, using the method described by Li et al. (2018).

**Table 1:** Demographic and clinical information on PD patients.

	Male	Female	*p*-value
Age	68.09 ± 6.29	68.00 ± 4.62	0.97
Total MDS UPDRS-III score	25.96 ± 11.16	27.90 ± 7.96	0.62
Sum of axial subscore	4.43 ± 2.25	3.90 ± 1.60	0.50
Sum of bradykinesia subscore	13.30 ± 6.83	13.60 ± 3.63	0.90
Sum of rigidity subscore	3.57 ± 2.95	2.50 ± 3.72	0.39
Sum of tremor subscore	4.65 ± 2.84	7.90 ± 2.96	**0.006**
H&Y	2.04 ± 0.77	2.00 ± 0.67	0.88
Disease duration (years)	8.04 ± 3.96	11.50 ± 7.17	0.08

Values are given as mean ± standard deviation. MDS UPDRS-III, Movement Disorder Society Unified Parkinson’s Disease Rating Scale-Part III; H&Y, Hoehn and Yahr stage. *p* < 0.05 is marked as bold.

### MRI data acquisition

**Figure 1:**
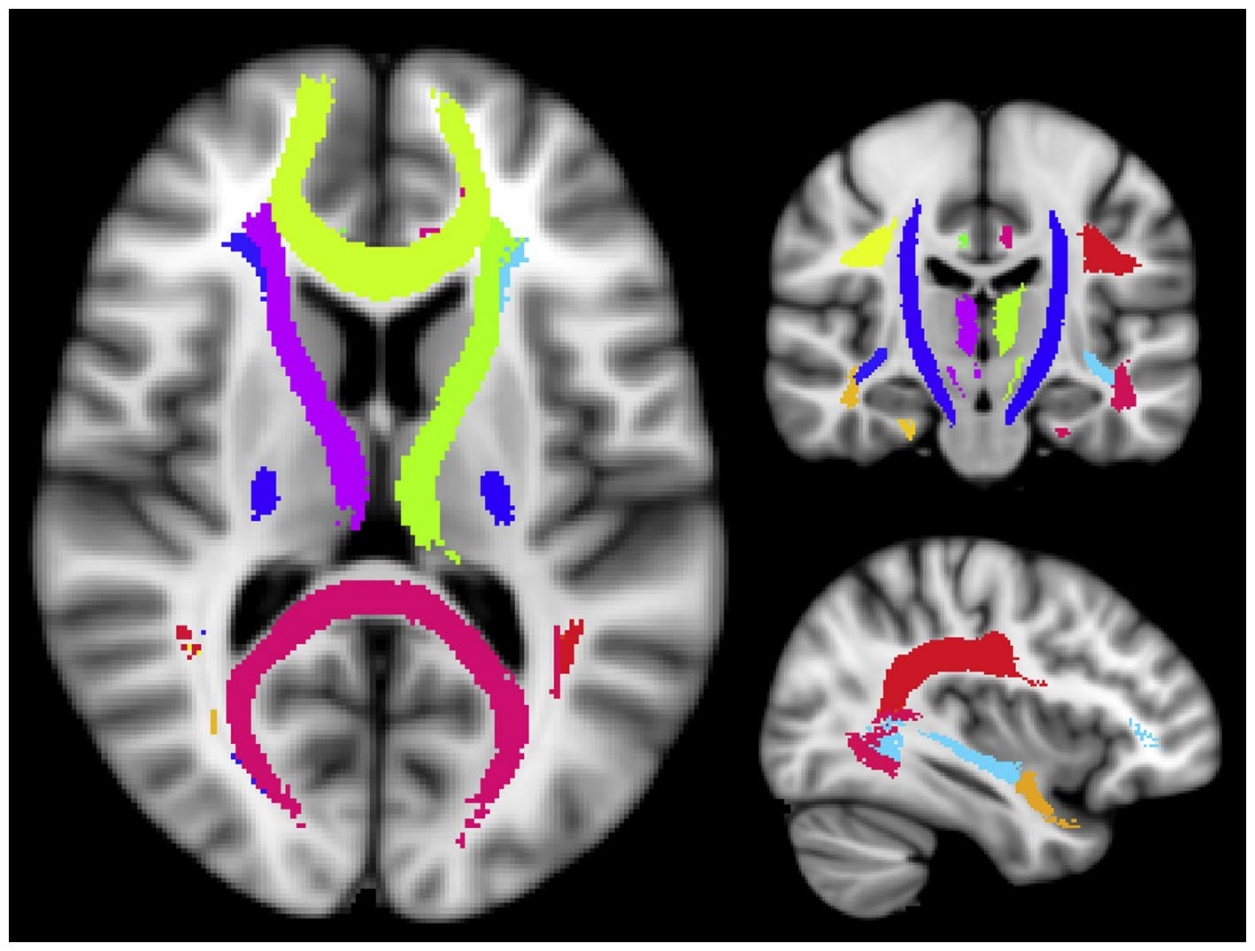
Visualization of the WM ROIs used in this study. The ROI names are listed in Table 2 (Tobias et al., 2019; Jiayue et al., 2022).

The subjects were scanned with a 3.0 Tesla MR scanner (Philips Achieva 3.0 Tesla; Philips Medical Systems, Netherlands) with an eight-channel head coil. We acquired a full brain 3DT1-weighted scan for structural references with an inversion recovery MPRAGE sequence TI = 808 ms, TR = 1800 ms, and an isotropic voxel size of 1 mm^3^. *T_2_* relaxation data were collected using a modified 3D GRASE sequence with 32 echoes with 10 ms echo spacing, TR = 1,000 ms, and the scanning time of 10.5 min. Twenty slices were acquired at 5 mm slice thickness and reconstructed to 40 slices at 2.5 mm. The in-plane voxel size was 1x1 mm. All subjects were scanned on the same day as their UPDRS assessment. The multiecho GRASE sequence was analyzed using in-house written MatLab (MathWorks, Natick, MA) code that uses a non-negative least squares (NNLS) fitting method to approximate the multiexponential decay curve with a number of exponential basis functions on a voxel by voxel level. The algorithm includes correction for stimulated echoes as well as a regularizer to make the fit more robust against noise in the time domain (Prasloski et al., 2012), resulting in one whole cerebrum MWF map per subject. We used the John’s Hopkins University WM tract atlas provided in the FSL package (Jenkinson et al., 2012) and registered them to each subject’s first echo of the T2 relaxation data before calculating the average MWF per region of interest (ROI). A total of 20 WM ROIs were used to cover the majority of the WM and delineate major WM tracts to get the most reasonable coverage for biological interpretation (Figure 1 and Table 2). All registrations were performed with the advanced normalization toolbox (ANTs) (Avants et al., 2008).

**Table 2:** Twenty white matter ROIs used in this study.

No	Name	No	Name
L1	Left Thalamic Radiation	R1	Right Thalamic Radiation
L2	Left Corticospinal Tract	R2	Right Corticospinal Tract
L3	Left Cingulum	R3	Right Cingulum
L4	Left Cingulum Hippocampus	R4	Right Cingulum Hippocampus
L5	Corpus Callosum Splenium	R5	Corpus Callosum Genu
L6	Left Inferior Fronto-Occipital Fasciculus	R6	Right Inferior Fronto-Occipital Fasciculus
L7	Left Inferior Longitudinal Fasciculus	R7	Right Inferior Longitudinal Fasciculus
L8	Left Superior Longitudinal Fasciculus	R8	Right Superior Longitudinal Fasciculus
L9	Left Uncinate	R9	Right Uncinate
L10	Left Arcuate	R10	Right Arcuate

### Statistical analysis

**Figure 2:**
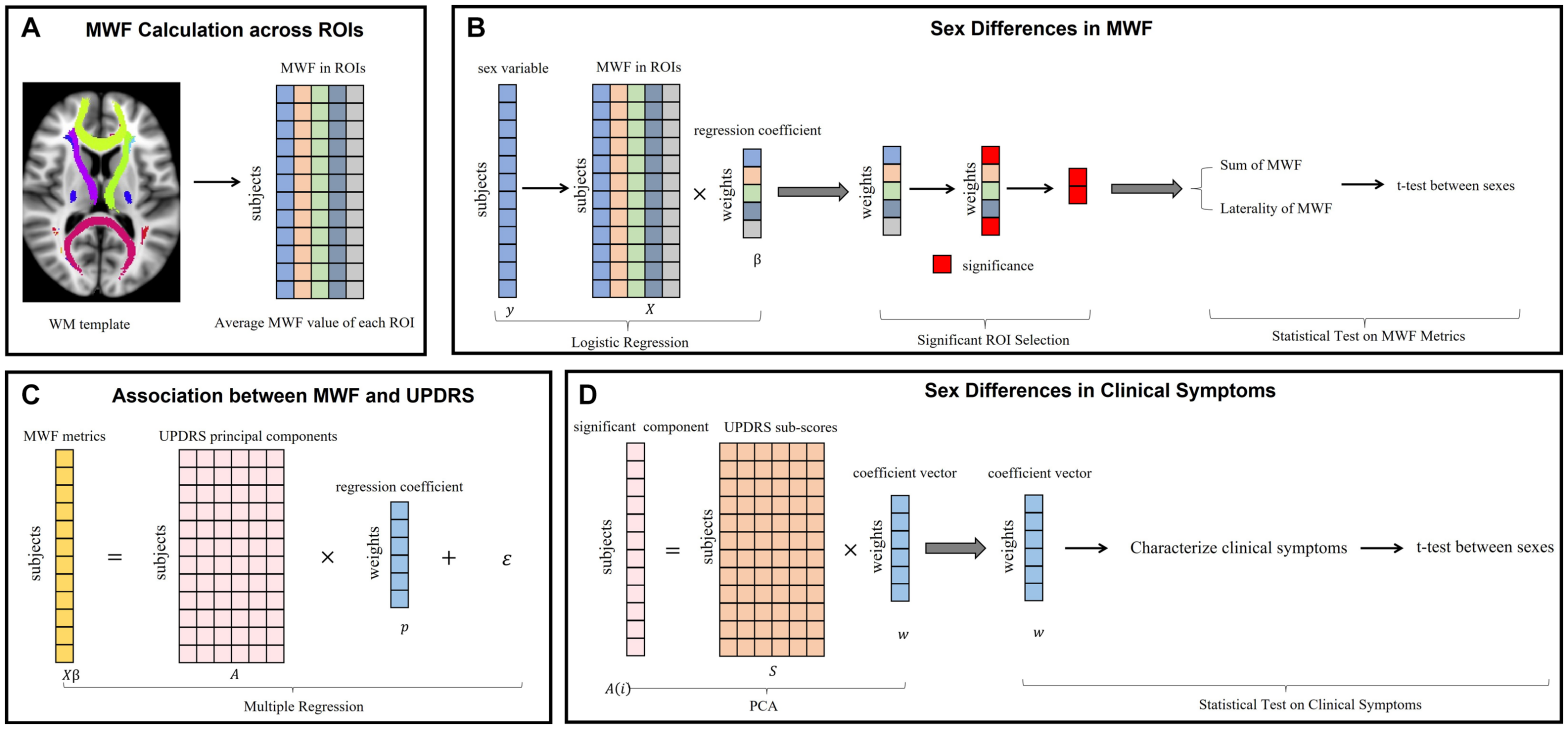
The overall workflow of the analyses in the study: **(A)** The average MWF in each ROI across all subjects is computed. **(B)** Assess sex differences in MWF. Logistic regression was performed, using the MWF values from panel **(A)** as the independent variables and binary-coded biological sex as the dependent variable. Significant weights were chosen to designate a subset of ROIs. The sum and laterality of MWF values in this subset were compared between males and females using *t*-tests. **(C)** Evaluate association between MWF and UPDRS. Principal Component Analysis (PCA) and multiple linear regression were used. **(D)** Assess sex differences in clinical symptoms.

The overall workflow of the analyses is shown in Figure 2. First, we calculated the average MWF of each white matter ROI (Figure 2A). Secondly, to assess sex differences in the myelin profiles, we performed logistical regression with sex as the dependent variable *y* and average MWF in each ROI as the independent variables *X* (Figure 2B). After assessing the coefficient vector *β* in the logistic regression model, we selected the subset of ROIs with significantly non-zero coefficients and then calculated the sum and laterality of MWF in this subset. A two-sample *t*-test was used to compare the differences between males and females in terms of these two MWF metrics.

To determine the parts of the UPDRS that corresponded to the sex differences in MWF, we performed multiple linear regression between UPDRS and MWF (Figure 2C). We first applied principal component analysis (PCA) on the different UPDRS motor subscores, with the optimal number of principal components determined by using cross-validation (Engemann and Gramfort, 2015) (which turned out to be 3). We then took the principal components of UDPRS motor scores as the independent variables *A*, and the linear combination of MWF values determined from the logistic regression as the dependent variable (i.e., *Xβ* from Figure 2B). After assessing the coefficient vector *p* in the multiple regression model, we examined whether or not there were principal components of UPDRS significantly associated with sex differences in MWF. Finally, we interrogated the weights (i.e., *w*) of individual UPDRS subscores (i.e., *S*) on the significant component (i.e., A(*i*), *i*th column of the matrix *A*) to evaluate the sex-related characteristics of clinical symptoms (Figure 2D). In addition, a two-sample t-test was used to examine the sex differences in clinical symptoms. Given the relatively small sample size, we further examined the robustness of our conclusions by using leave-one-out cross-validation. A false discovery rate (FDR) correction was applied for multiple comparisons. Statistical significance was set at *p* < 0.05.

**Figure 3:**
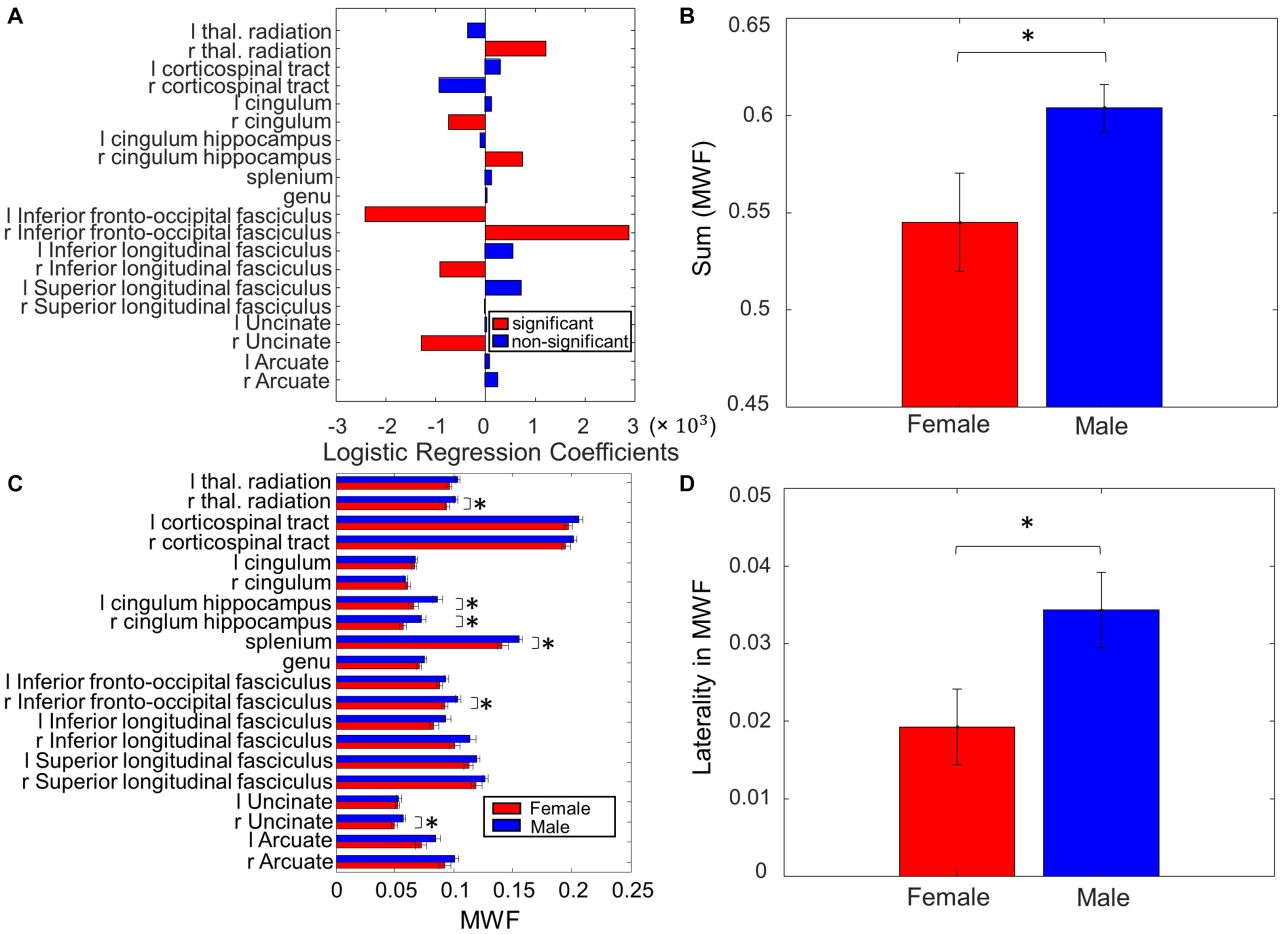
Sex differences in MWF. **(A)** Coefficients on MWF ROIs in the logistic regression to predict sex. **(B)** The total MWF value in the significant ROIs in **(A)** exhibited significant differences between Male and Female. **(C)** Comparison of the MWF value for each ROI between Male and Female. **(D)** Contrasting the left and right-sided MWF in the significant ROIs in **(A)** resulted in significant differences between Male and Female (*p < 0.05, **p < 0.01).

## Results

### Demographic and clinical characteristics

Patients were at relatively mild stages of disease (H&Y stage 2) of moderate duration. Mean age, H&Y disease severity, and disease duration were not significantly different between male and female patients (Table 1). Male and female patients also did not significantly differ in total MDS UPDRS-III score, nor motor subscores of axial symptoms, bradykinesia, or rigidity. However, female patients had significantly higher tremor scores than male patients (*p* = 0.006).

### Sex differences in myelin

When assessing sex differences in MWF using logistic regression, we found MWF of the following ROIs significant in predicting sex: thalamic radiation, cingulum, cingulum hippocampus, inferior fronto-occipital fasciculi (IFOF), inferior longitudinal fasciculi (ILF), and uncinate (*p* < 0.05, Figure 3A). The total MWF of the aforementioned significant ROIs was significantly higher in males compared to females (*p* = 0.02, Figure 3B). When comparing the MWF of each ROI individually, males appeared to have greater MWF than females across all ROIs, and this was significant (uncorrected) in the following ROIs (Figure 3C): R thalamic radiation (*p* = 0.03), L cingulum hippocampus (*p* = 0.02), R cingulum hippocampus (*p* = 0.02), splenium (*p* = 0.04), R IFOF (*p* = 0.02), and R uncinate (*p* = 0.04). Overall, the total MWF was larger in right-sided ROIs in both males (*p* = 0.006) and females (*p* = 0.07, not significant, but a trend). We further examined this laterality of MWF by taking the absolute difference between right and left-sided MWF among the significant ROIs (Figure 3A) and found that males had greater laterality of MWF (*p* = 0.03, Figure 3D).

### Sex differences in UPDRS motor scores

**Figure 4:**
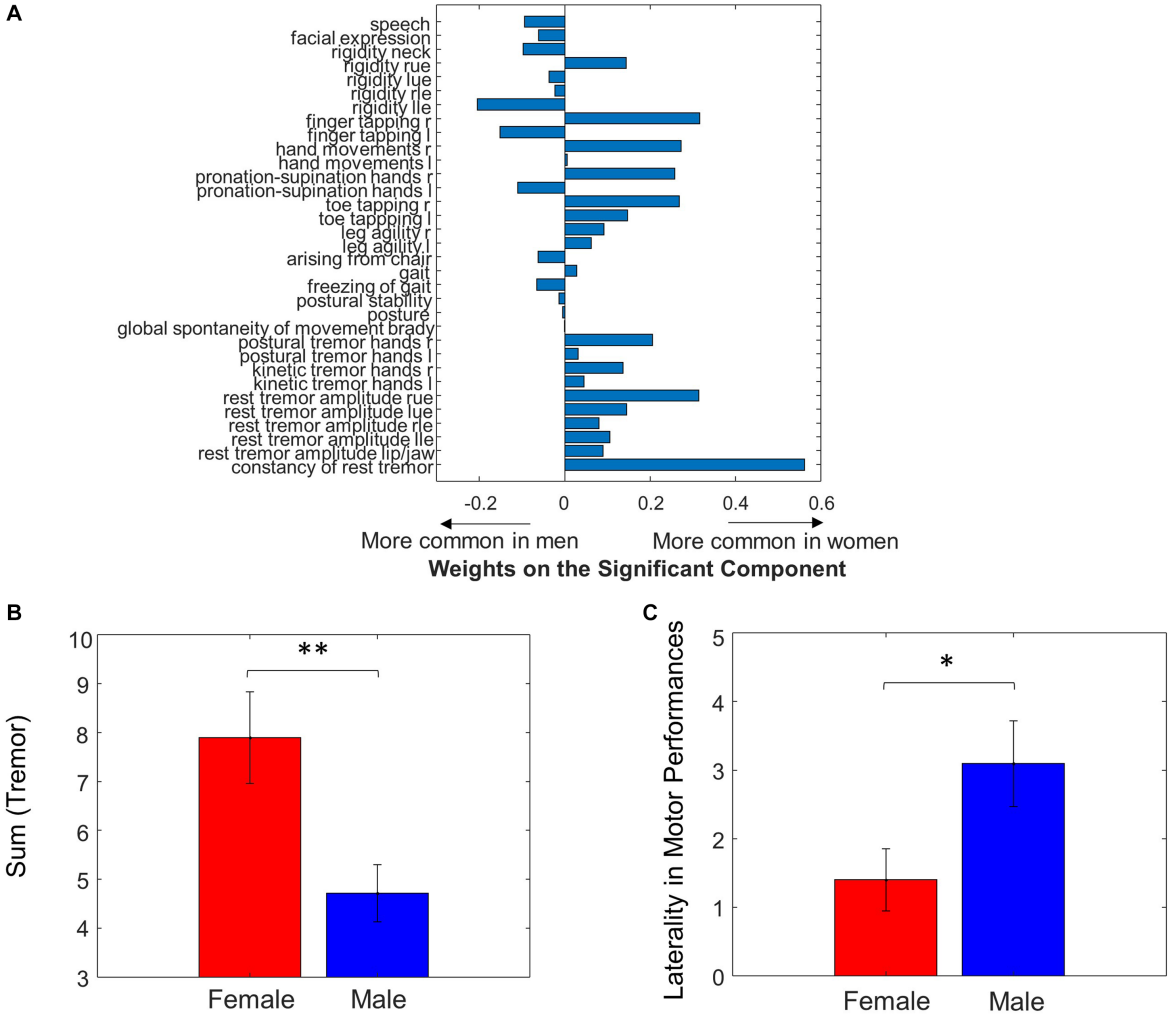
Sex differences in UPDRS motor scores. **(A)** Weights of individual UPDRS motor scores on the significant principal component that predicted MWF values. **(B)** Summing the tremor scores resulted in significant differences between Male and Female. **(C)** Laterality between left and right side in motor performance was different between sexes (*p < 0.05, **p < 0.01).

When assessing the correspondence between UPDRS and the sex differences in MWF using multiple linear regression, the second principal component of the UPDRS score was significant (*p* = 0.02) in predicting the sex differences in MWF. The weights of this principal component are shown in Figure 4A. Note that the weights related to tremor and bradykinesia favored females, and weights related to rigidity and axial symptoms favored males. This was partially reflected in the raw data, where females had significantly greater total tremor score than males (*p* = 0.006, Table 1 and Figure 4B). However, there were no statistically significant differences in bradykinesia, rigidity, and axial symptom subscores between males and females. Weights related to motor performance (i.e., finger tapping, hand movements, pronation-supination hands, toe tapping and leg agility) tended to be different between left and right. Overall, the total motor performance score was larger (indicating greater symptom severity) on the left side in both males (*p* = 0.04) and females (*p* = 0.17, not significant, but a trend). We further examined this laterality of motor performance scores, by taking the absolute difference between all left and right-sided motor performance scores, and found that males had greater laterality of motor performance scores (*p* = 0.04, Figure 4C).

**Figure 5:**
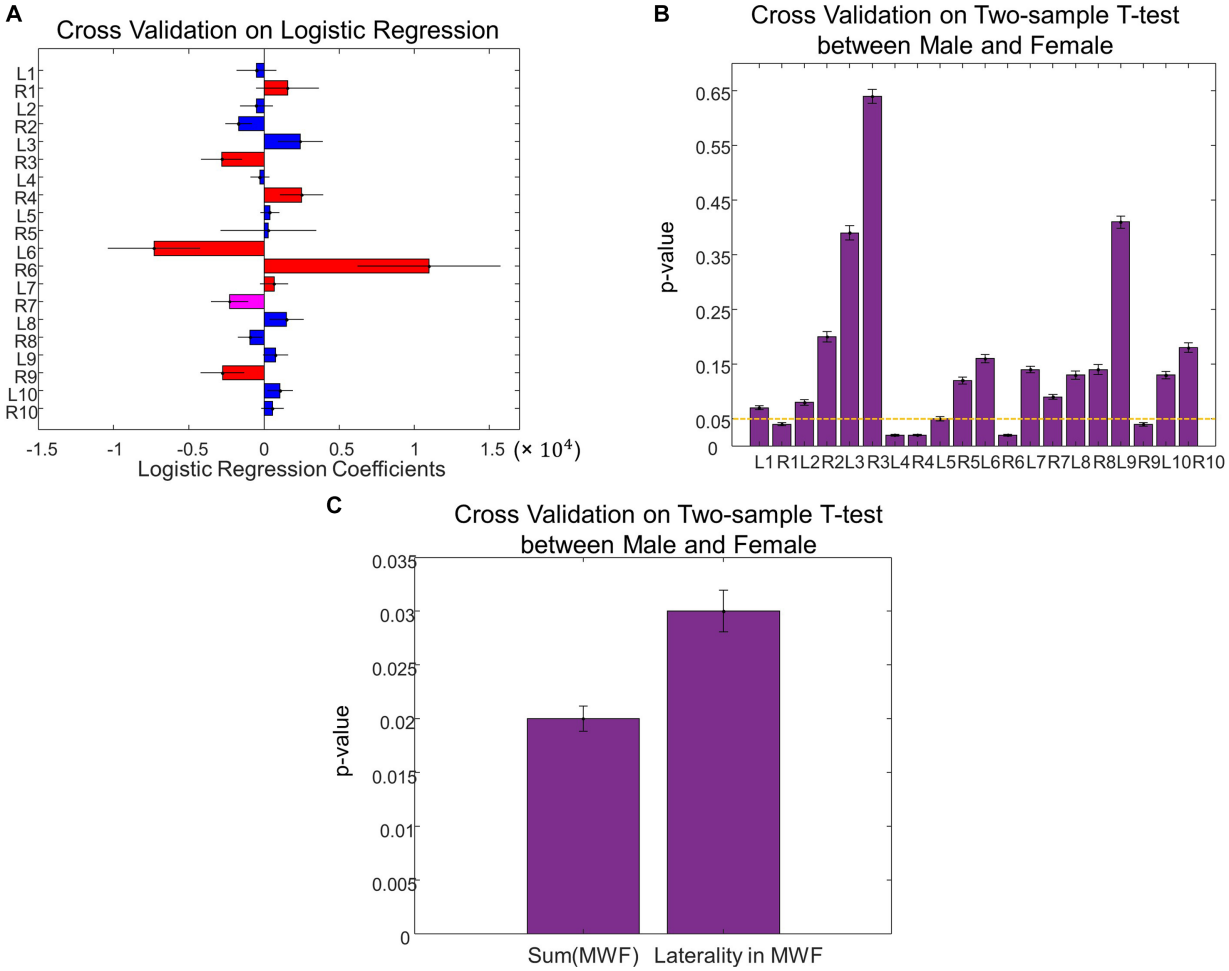
Cross validation analysis on sex differences in MWF. **(A)** Leave-one-out cross validation coefficients on MWF ROIs in the logistic regression to predict sex. **(B)** Comparison of the MWF value for each ROI between Male and Female using the leave-one-out cross validation. **(C)** The total MWF and the laterality of MWF exhibited significant differences between Male and Female using the leave-one-out cross validation.

**Figure 6:**
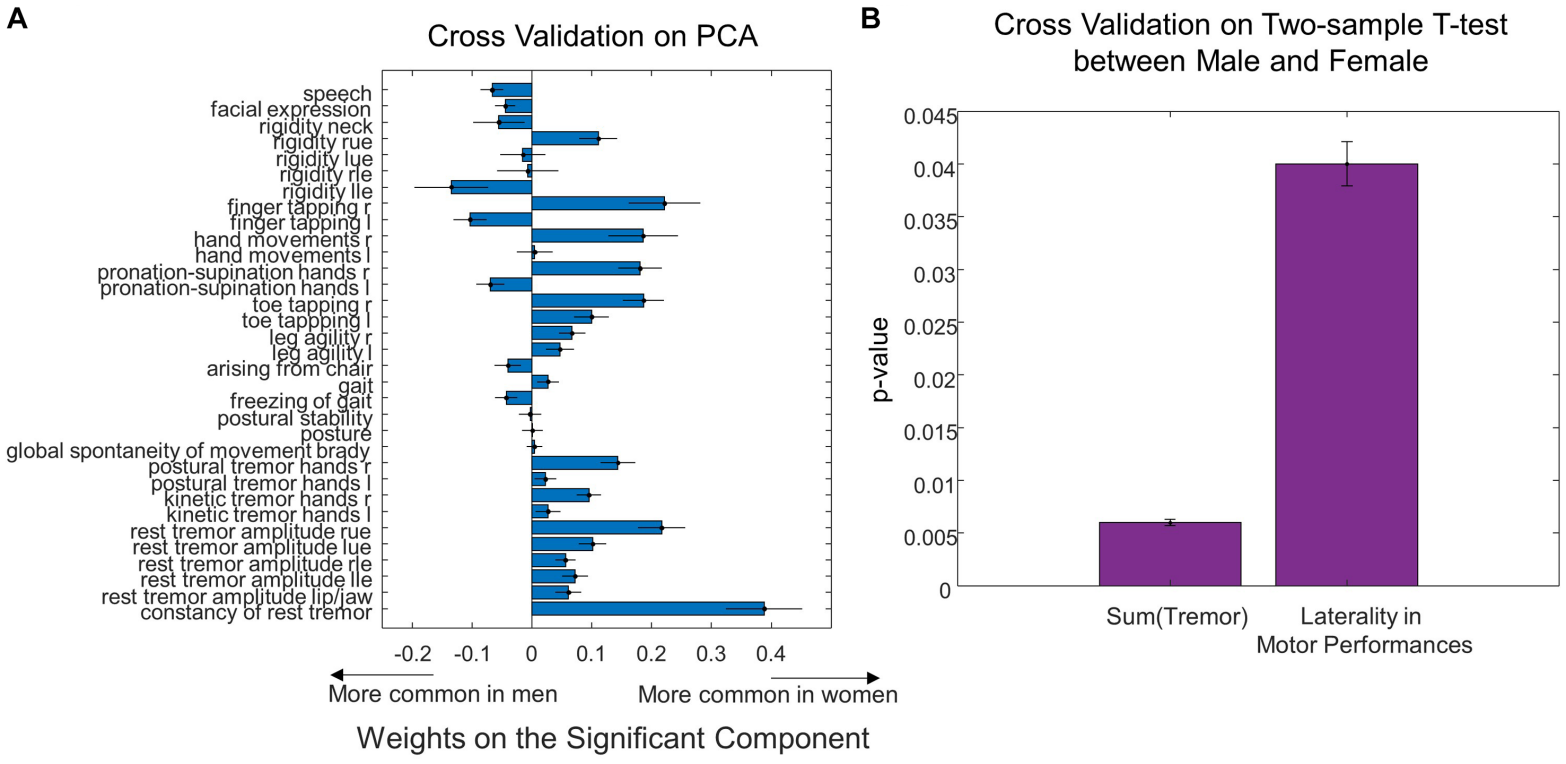
Cross validation analysis on sex differences in UPDRS motor scores. **(A)** Leave-one-out cross validation weights of individual UPDRS motor scores on the significant principal component that predicted MWF values. **(B)** The total tremor score and the laterality in motor performances exhibited significant differences between Male and Female using the leave-one-out cross validation.

Using leave-one-out cross-validation, we found the MWF ROIs that were most frequently selected as significant in predicting sex were unchanged. While we found seven significant MWF ROIs using the whole dataset (Figure 3A), the top seven MWF ROIs in the leave-one-out cross-validation were the same except for the shift from right ILF (the eighth most significant ROI in the cross-validation analysis) to left ILF (Figure 5A). Sex differences in the individual/total MWF and the laterality of MWF remained significant in the cross-validation analysis (Figures 5B,C). Similar to the whole dataset analysis, we found the significant principal component of UPDRS scores (*p* = 0.03 ± 0.01) in predicting sex differences in MWF in the cross-validation analysis. The weight distribution of the significant principal component in the cross-validation analysis (Figure 6A) was very similar to that in the whole dataset analysis (Figure 4A). Sex differences in the total tremor score and the laterality of motor performance, remained significant in the cross-validation analysis (Figure 6B).

## Discussion

Our findings of sex-related WM changes that are associated with sex differences in clinical presentation contributes to the increasing body of knowledge regarding the role of biological sex in PD. Biological sex has an important modulating effect on the clinical features of PD, evidenced by well-established epidemiological differences in prevalence (Miller and Cronin-Golomb, 2010), symptom profile (Baba et al., 2005; Haaxma et al., 2007; Colombo et al., 2015), and natural progression (Jurado-Coronel et al., 2018). To the best of our knowledge, this study is the first of its kind to study sex differences in myelin and its relation to clinical presentation in PD.

### Sex differences in UPDRS motor scores

We found the principal component of UPDRS scores predicting sex differences in MWF had tremor and bradykinesia weights favoring females, and rigidity and axial symptom weights favoring males (Figure 4A). This demonstrates that the sex-specific differences in motor performance were associated with sex-specific differences in myelination. When looking at the raw clinical data, this observation was supported by noting female patients had significantly higher tremor scores compared to male patients (*p* = 0.006, Table 1).

The tremor-dominant phenotype of PD is classically regarded as a more benign subtype of PD, whereas rigidity and postural instability-dominant phenotypes have been regarded as having poorer prognosis. Clinical presentations are key features for different subtypes of diseases (Thenganatt and Jankovic, 2014; Wang et al., 2021). Female sex has been associated with the tremor-dominant phenotype and slower disease progression in prior studies (Haaxma et al., 2007; Solla et al., 2012). Female patients are also noted to have older age of disease onset, which has been positively associated with factors increasing cumulative estrogen exposure such as higher parity, later age of menopause, and longer fertile life span (Haaxma et al., 2007), suggesting a role for sex hormones in disease onset, clinical characteristics, and progression. While we did not observe a significant difference in the axial, rigidity, and bradykinesia subscores between male and female patients in the raw data, the weights related to rigidity and axial symptoms were biased toward males in the principal component that was significant in predicting sex differences in MWF.

### Sex differences in myelin

The MWF of the thalamic radiation, cingulum, cingulum hippocampus, IFOF, ILF, and uncinate were significant in predicting sex (Figure 3A). Males had higher overall MWF values (Figure 3B), and specifically higher MWF in the R thalamic radiation (*p* = 0.03), L cingulum hippocampus (*p* = 0.02), R cingulum hippocampus (*p* = 0.02), splenium (*p* = 0.04), R IFOF (*p* = 0.02), and R uncinate (*p* = 0.04), compared to females. Sex differences in myelin in the PD population have yet to be studied. However, there is emerging evidence that sex hormones play a significant role in the sexual differentiation of WM microstructure, which may be driven by differences in myelin (van Hemmen et al., 2016). van Hemmen et al. compared WM microstructure in karyotype XY men, karyotype XX women, and karyotype XY women with complete androgen insensitivity syndrome (CAIS), and found that karyotype XY women with CAIS showed female-typical FA throughout many WM ROIs, which was predominantly due to female-typical RD. This suggests a significant effect of sex hormones on human neural sexual differentiation, and specifically on WM and myelin microstructure. Male participants of this study had lower RD (suggestive of greater myelination) in the left internal and external capsule, corona radiata, cerebral peduncle, uncinate, superior fronto-occipital fasciculus, pallidum, and amygdala, compared to female participants. Other studies have also found diffusion changes suggestive of sex differences in myelin. Similar to the results of our study, Menzler et al. found higher values of FA and lower RD in the thalamus, corpus callosum, and cingulum of male subjects compared to females. Jung et al. found that males had lower RD in the left anterior thalamic radiations and left uncinate fasciculus when compared to female subjects. However, most existing studies of sex differences in WM, including the above mentioned, are in the young, healthy adult population. In order to determine sex-specific myelin changes in PD, more studies of sex differences in myelin in the healthy elderly population are needed. Inano et al. examined the effects of age and sex on FA, axial diffusivity (AD), and RD in WM, in a large study of 857 healthy subjects with a wide age range (25–85, mean age 56, 32). Global FA was negatively correlated with age, whereas global RD was strongly positively correlated with age, suggesting that myelin degeneration was implicated in age-related changes in WM integrity. Interestingly, this study did not find a significant interaction between age and sex, suggesting that there are no sex differences in the aging process of WM. The WM ROIs identified in our study have some overlap with previously noted sex differences in myelin among the healthy young adult population. This suggests some of the observed differences may be in part due to normal sexual dimorphism in WM, and some due to PD-specific differences in myelin. Unlike the healthy elderly participants studied by Inano et al. (2011), we found sex differences in myelin profiles among our study population with PD, which may reflect PD-specific sex differences. However, due to the overall paucity of studies investigating sex differences in myelin, it is difficult to differentiate which of the observed ROIs are representative of normal sexual dimorphism of myelin, versus PD-specific sexual dimorphism of myelin. Given that sex differences in MWF observed in our study were associated with previously established sex differences in clinical symptom profile (tremor-dominant phenotype in females, rigidity and postural instability phenotype in males), we suggest that there is a role of myelin differences in clinically-observed sex differences in PD.

### Laterality of MWF and motor performance scores

Clinically, PD is well-recognized as an asymmetric disease, with a characteristic unilateral onset of motor symptoms, associated with more severe nigrostriatal degeneration on the contralateral side. Claassen et al. described a “left hemisphere susceptibility” in PD, which persists after accounting for side of disease onset as well as handedness (Claassen et al., 2016). The results of the present study may add to this previous notion of left-sided susceptibility in PD. We found that MWF was larger in right-sided ROIs (indicating more myelination on the right) in both males (*p* = 0.006) and females (not significant, but a trend). This laterality of myelination was greater in males when taking the absolute difference between right and left-sided ROIs (*p* = 0.03, Figure 3D). Motor performance scores were also larger (indicating greater symptom severity) on the left side in both males (*p* = 0.04) and females (not significant, but a trend), and again, males had greater left-sided laterality of motor performance scores (*p* = 0.04, Figure 4C).

### Role of sex hormones in PD

The mechanisms of sex differences in PD have not yet been fully elucidated, but a key role of sex hormones is likely. Estrogen has been associated with neuroprotective effects on the nigrostriatal dopaminergic system, with various proposed mechanisms, including inhibition of reactive glia, inhibition of the inflammatory cytokine cascade, antioxidative effects, and anti-apoptotic effects. Early menopause, whether natural or surgically induced, is associated with increased risk of parkinsonism, and the risk is increased with younger age at oophorectomy (Rocca et al., 2008). Neuroprotective effects of estrogen have also been observed in mouse models with dopaminergic striatal neurotoxicity induced by 1-methyl-4-phenyl-1,2,3,6-tetrahydropyridine (MPTP) (D’Astous et al., 2004). However, there are also studies that have found the opposite association between estrogen and PD, or even a lack of association (Smith and Dahodwala, 2014). Given there are complex interplays in biological systems, as well as differential effects of hormones depending on how (ex. cyclical vs. continuous), when (ex. pre vs. post-menopausal), and in what form they are administered (stereoisomers of estradiol, estriol), definitive conclusions regarding estrogen and clinically observed sex differences PD are difficult to establish.

Sex-specific differences in clinical characteristics, with a predilection toward a more benign clinical phenotype in female PD patients, may support the neuroprotective role of estrogen. The effects of estrogen on myelination and cellular pathology in PD is unclear. However, given robust clinical differences in PD between male and female patients, these differences may in part be explained by the effects of sex hormones. In our study, though females had the more benign tremor-dominant phenotype, male PD patients had overall higher MWF values, and specifically in the R thalamic radiation, bilateral cingulum hippocampus, splenium, R IFOF, and R uncinate, indicating more myelination in these WM ROIs.

In this study, we used MWF instead of DTI metrics to assess myelin integrity in PD. We have previously shown that using DTI measures to define ROIs, but then interrogating the MWF values within these ROIs is more sensitive and specific than DTI for predicting biological sex in healthy controls (Baumeister et al., 2020). Finally, we have previously shown that white matter profiles assessed via MWF were able to predict clinical subtypes of PD, but FA was not (Baumeister et al., 2019). Thus, based on our prior work demonstrating superiority of MWF in predicting sex and clinical aspects of PD, we felt justified in using MWF for looking at the relationships between sex differences and myelin and PD.

### Limitations

There are a number of limitations to our study. Our sample size is relatively small. Myelin water imaging is a relatively new sequence that does not have the same availability as other more popular, but more qualitative, sequences such as FLAIR. However, even with these relatively small numbers we observed statistically significant results. The number of subjects was not balanced between the sexes, but we note that our sample has roughly the same 2: 1 male to female ratio as the incidence of the disease (Miller and Cronin-Golomb, 2010). While we found relatively broad overall changes in myelin between the sexes; a larger sample size may determine if changes in particular ROIs are associated with specific clinical features.

We have implicitly implied a causal direction between changes in myelin and clinical presentation. While this seems likely, within the limitations of cross-sectional data it is difficult to establish that the observed sex differences in MWF of WM ROIs caused differences in clinical characteristics of male and female PD patients. We cannot discount that other factors might jointly affect myelin and clinical features. Moreover, we cannot discount that physical activity induces plasticity in myelin (Fields, 2015), and thus causal directions between clinical features and myelin may become less clear.

The cerebellum and cerebello-thalamo-cortical pathway are frequently postulated as contributors to tremor genesis in PD and essential tremor patients, but the cerebellum was excluded in the WM ROI atlas. While we took a whole-brain network-perspective and identified widespread changes in WM microstructure, we cannot exclude specific changes in the cerebellum because the myelin water imaging sequence employed does not include the cerebellum. This might be corrected as the MWF technique matures and becomes more widespread.

### Conclusion

Sex differences are apparent in the clinical characteristics of PD. Recent evidence suggests a potential role for myelin changes in the cellular pathology of PD, as well as in driving sex differences in WM microstructure in healthy adults. This necessitates sex-specific investigation of microstructural and cellular pathology in PD. In this study, we found sex differences in myelin associated with sex differences in UPDRS motor symptom profile. While preliminary, our results suggest that further investigation of the role of biological sex in myelin pathology and clinical presentation in PD is warranted.

## Data availability statement

The raw data supporting the conclusions of this article will be made available by the authors, without undue reservation.

## Ethics statement

The studies involving humans were approved by the Institutional Review Board of University of British Columbia. The studies were conducted in accordance with the local legislation and institutional requirements. The participants provided their written informed consent to participate in this study.

## Author contributions

JC and JK contributed to the methodology and writing of this project. JC, YW, TB, and AL contributed to the analysis of data. JC and YW conducted the result validation. JK, MZ, and SL contributed to the acquisition of data. MM and JK performed the interpretation of data. JC, JK, YW and MM contributed to the conceptualization of this project. MM and YW performed the review and editing of the manuscript. JC, YW and MM contributed to the funding acquisition. All authors contributed to the article and approved the submitted version.

## Funding

This work was in part supported by National Natural Science Foundation of China (Grant No. 62201357), Shenzhen Science and Technology Program (Grant Nos. RCBS20221008093347105 and 20220812132050001), Shenzhen University (SZU) Top Ranking Project (Grant No. 86000000210), and the John Nichol Chair in Parkinson’s Research.

## Conflict of interest

The authors declare that the research was conducted in the absence of any commercial or financial relationships that could be construed as a potential conflict of interest.

## Publisher’s note

All claims expressed in this article are solely those of the authors and do not necessarily represent those of their affiliated organizations, or those of the publisher, the editors and the reviewers. Any product that may be evaluated in this article, or claim that may be made by its manufacturer, is not guaranteed or endorsed by the publisher.
